# The largest bilateral gemination of permanent maxillary central incisors: Report of a case

**DOI:** 10.4317/jced.51197

**Published:** 2013-12-01

**Authors:** Abbas Shokri, Maryam Baharvand, Hamed Mortazavi

**Affiliations:** 1 Assistant professor, Department of Oral and Maxillofacial Radiology, Dental School, Hamadan University of Medical Sciences, Hamadan, Iran; 2 Associate professor, Department of Oral Medicine, Dental School, Shahid Beheshti University of Medical Sciences,Tehran, Iran

## Abstract

Gemination is defined as an attempt to make two teeth from one enamel organ. Bilateral presentation of this phenomenon is very rare, with prevalence of 0.01% to 0.04% in the primary, and 0.05% in the permanent dentition. This paper describes a rare case of huge bilateral gemination of permanent maxillary central incisors in a nine-year-old Iranian boy with poor aesthetic. The patient did not have history of anomaly in his primary dentition and in his family either. This type of dental anomaly can cause clinical problems in the form of malocclusion, poor aesthetic, and impaction of adjacent teeth, caries, and periodontal destruction.

** Key words:**Gemination, central incisor, bilateral.

## Introduction

Variation in the number, form and size of teeth may occur in primary and permanent dentition. The terms double tooth, double formation, linking tooth, fused teeth, jointed tooth, dichotomy, connation, dental twining, synodontia and schizodontia, mirror-image double tooth, and geminated composite odontoma are often used to describe fusion or gemination, both of which are primary developmental abnormalities of the teeth (1,2). It is generally accepted that gemination originates when one tooth bud attempts to split into two, while fusion results from the conjoining of two tooth buds (3).

The prevalence of double teeth in the primary and permanent dentition ranges from 0.4% to 0.9 % and 0.1% to 0.2 %, respectively (4,5). Geminated teeth are mostly unilateral, so that bilateral presentation of this phenome-non is very rare with the prevalence of 0.01% to 0.04% in the primary, and 0.05% in the permanent dentition (6). There is no sex predilection and geminated teeth are usually found in the maxilla, while cases of fusion are more frequently seen in the mandible (4,5).

Gemination and fusion are generally asymptomatic. However, teeth can cause clinical problems in the form of malocclusion, poor aesthetic, impaction of adjacent teeth, caries or periodontal destruction (2,6).

In fact, the co-operation of practitioners with expertise in different fields of dentistry is important to create or achieve functional and aesthetic success in these patients. Several treatments such as endodontic, restorative, surgical, periodontal and/or orthodontic procedures have been described in the literature with respect to the different morphological variations of double teeth (6).

This paper reports a rare case of huge bilateral gemination of permanent maxillary central incisors in a ni-ne-year-old Iranian boy which caused crowding.

## Case report

**Figure 1:**
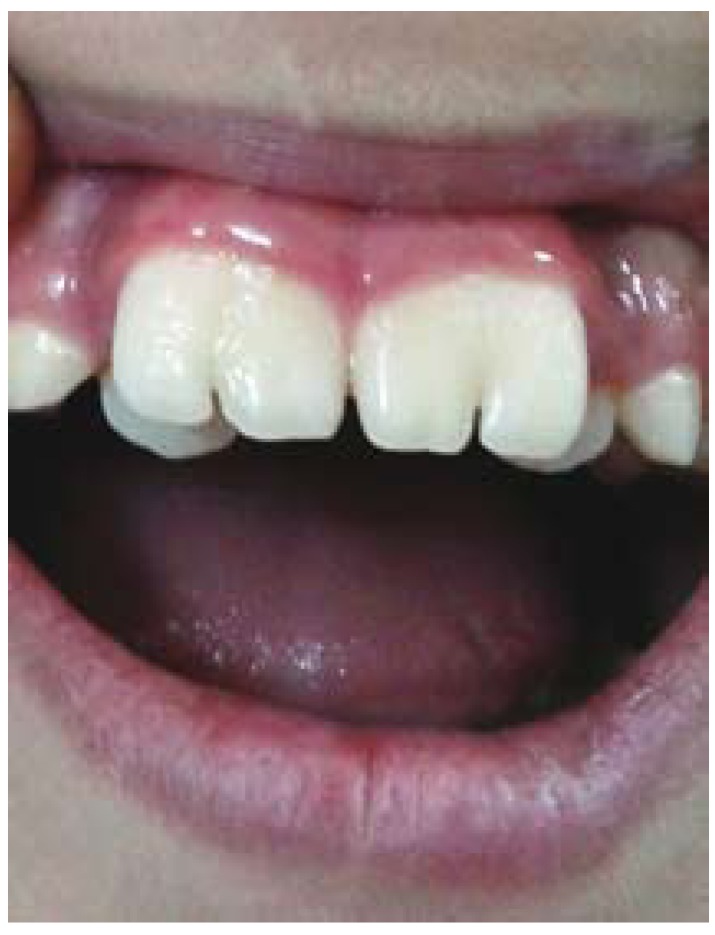
Clinical view of bilateral geminated permanent maxillary central incisors with deep notches.

A nine-year-old boy was referred to the Department of Oral and Maxillofacial Radiology for the evaluation of enlarged permanent maxillary central incisors, which caused aesthetic and chewing problems (Fig. 1).

There was neither remarkable medical history nor family history of dental anomalies.

Intraoral examination revealed permanent central maxillary incisors, with the width of 12.4 mm in the right central incisor and 12.8 mm in the left central incisor, which had incisal notches, with depth of 2.66 mm and 4.76 mm in the right and left maxillary incisors, respectively. Thermal pulp testing, percussion and periodontal probing showed no abnormalities. The patient was in the mixed dentition and the number of teeth was normal.

**Figure 2:**
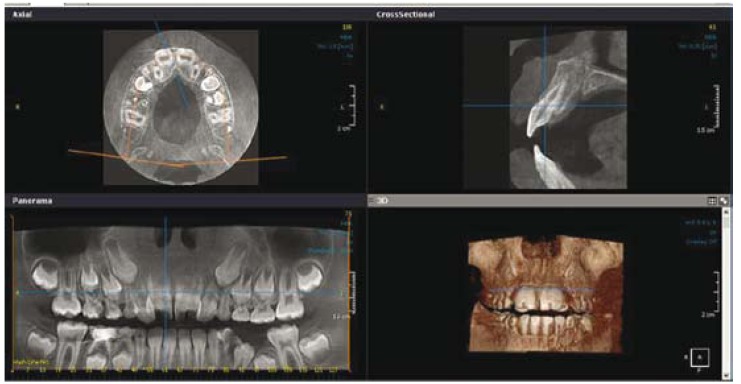
CBCT shows geminated central incisors in panorama, cross sectional, axial, and 3D views.

The space between primary maxillary left and right canines had been completely filled by enlarged teeth so that both lateral incisors were also palataly displaced. For radiographic evaluation, cone beam computed tomography (CBCT) was taken (Fig. 2).

Each maxillary permanent central incisor had a single rood with two separate canals with one orifice in the apex. On the basis of clinical and radiographic findings, diagnosis of bilateral geminated permanent central incisors was made and orthodontic and operative treatments were planned. The treatment plan was explained to the patient’s family, but they could not afford any treatment plan.

## Discussion

Gemination is the result of a developmental aberration of both the mesoderm and the ectoderm. These disturbances are related to the local metabolic interferences occurring during morpho differentiation of the tooth germ. The main etiology of gemination remains unknown, but physical pressures leading to the union of teeth and genetic inheritance have been suggested as possible causes (2,4).

It is hard to differentiate between gemination and fusion. Some researchers tried to differentiate them by counting the teeth. Gemination is defined as a single enlarged tooth in which the tooth count is normal. However, fusion is defined as a single enlarged tooth in which the tooth count reveals a missing tooth. Moreover, gemination and fusion are being used as synonyms as well. Finally, some authors simply call the phenomenon as “connected teeth” or “double teeth” to avoid confusion over terminology (3,7).

Gemination in permanent dentition is a rare phenomenon with the prevalence of 0.1 -0.2%. It is mostly unilateral so that its bilateral presentation is extremely rare with the prevalence of 0.01 to 0.05% (4,5). Türkaslan et al and Sener et al reported few cases of this type of gemination (6,8).

According to Nik-Hussein, the anomalies of permanent teeth are strongly associated with anomalies in the primary dentition; for example, presence of gemination in primary dentition is associated with involvement of permanent teeth in approximately 60% of cases (7). However, in our case the patient did not have any dental abnormalities in his primary dentition with no evidence of the same dental problems in his family members.

Chipashvili et al pointed out that maxillary central incisor are most commonly affected by gemination. This finding is in agreement with our case (3). Grover et al reported geminated teeth in lateral incisors, canines, premolars and molars (2).

Grammatopoulos et al showed that gemination could be seen in both sexes with equal frequency (5). Grover et al and Sener et al reported the same finding as well (2,8).

According to Aguiló et al, there is no difference in the proportion of double teeth in either the maxilla or mandible (9). Neves et al reported involvement of mandibular teeth (4). In contrast, Brook et al demonstrated that gemination is usually found in the maxilla (10). In our case maxillary teeth were affected.

Clinical presentation of geminated teeth varies from a minor notch in the incisal edge of the affected tooth to the appearance of almost two separate crowns (5). In our case, incisal notch was seen in both geminated teeth. According to available data, our case is the largest bilateral geminated permanent maxillary central incisors reported ever. Sener et al reported bilateral geminated teeth which were more similar to our case in terms of size (8).

Diagnosis of double teeth is accomplished based on clinical and radiological examinations. According to Sener et al, conventional dental radiographs are not usually sufficient to establish a proper diagnosis (8).

Therefore, computerized tomography (CT) is suggested (8). Our patient was evaluated by cone beam computed tomography (CBCT) to make an accurate diagnosis accordingly.

Unfortunately, our patient’s parents could not afford any treatment plan.
